# A Pediatric Appropriateness Evaluation Protocol for Iran Children Hospitals

**DOI:** 10.5812/ircmj.16602

**Published:** 2014-07-05

**Authors:** Anvar Esmaili, Hesam Seyedin, Obeidollah Faraji, Jalal Arabloo, Yaghoub Qahraman Bamdady, Shahin Shojaee, Saadat Hesam

**Affiliations:** 1 Department of Health Management and Economics, School of Public Health, Tehran University of Medical Sciences, Tehran, IR Iran; 2 Health Management and Economics Research Centre, School of Health Management and Information Sciences, Iran University of Medical Sciences, Tehran, IR Iran; 3 Mahabad Imam Khomeini Hospital, Urmia University of Medical Sciences, Urmia, IR Iran

**Keywords:** Reliability, Validity, Iran, Hospital

## Abstract

**Background:**

Applying utilization review programs is an appropriate solution to decrease the expenditure, and to increase the efficiency of healthcare systems.

**Objectives:**

This paper presents an instrument to measure the level of appropriate admissions and days of stay (DOS) in the pediatric public hospitals of Iran.

**Materials and Methods:**

The American version of the Pediatric Appropriateness Evaluation Protocol (PAEP) was modified and adjusted by our group of physicians. They carried out a retrospective study over 100 randomly selected patients. The reliability of the instrument was tested based on the consensus of reviewers using PAEP. In addition, the external validity of the instrument was studied by comparing the evaluations of the reviewers using PAEP and the individual judgments of three clinicians in two public teaching hospitals. Finally, reliability and validity were also calculated by the kappa statistic.

**Results:**

With respect to the inter-reliability testing, there was a high level of agreement between reviewers applying the instrument in the admissions criteria and days of stay. Overall agreement was > 77%; also specific inappropriate agreement and specific appropriate agreement were > 61%, and > 72%, respectively. Regarding the validity of the testing, the instrument had a sensitivity of > 0.75, specificity of > 0.67, as well as positive and negative predictive values of > 0.93, and > 0.55, respectively. The kappa statistic for the reviewers (using the instrument for admission and days of stay criteria) were substantial (k = 0.75.5 and 0.71). They were also substantial for clinicians (k = 0.73 and 0.66).

**Conclusions:**

These results showed that the modified PAEP is a reliable and valid instrument to study the appropriateness of admission and days of stay in Iran hospitals. As the developing countries, particularly, Middle East countries have the same status and culture, the result of this study (with minor changes) could be applied in these countries too.

## 1. Background

Efficient and cost-effective use of resources is very important for countries such as Iran where resources allocated to the health care system are limited. The total health expenditure (as % of GDP) in Iran was reported at 5.60 in 2010 (1). In Iran, however, hospital expenditure raised more than three times from 2002 to 2007 (2). Although the costs of health care in Iran are much lower than developed countries, concerns regarding the rising expenditures and limited efficiency of hospitals are increasing (3). Given, the inappropriate admissions in some hospitals, the usefulness of utilization review instruments seems quite clear in order to decrease the cost (4). In addition, based on the methods of payment (fee for service) and their effects on increasing bed occupancy in Iranian hospitals (5), this instrument can be helpful in limiting the demand and controlling the costs of the services too.

The implementation of these programs presents a practical solution to the problems of increase in cost and lack of efficiency. Therefore, such an implementation must be based on a practical method, which is both reliable and valid. One of the most extensively used utilization tools for assessing pediatric admissions and days of stay (DOS) is the Pediatric Appropriateness Evaluation Protocol (PAEP). Kreger and Restuccia (6) modified this tool from its adult version (7). Werneke et al. (8) in their study found that the North American PAEP had limited validity for evaluating British pediatric admissions as well as DOS and concluded that utilization review instruments developed in one health system may not be transferable to another.

## 2. Objectives

This paper illustrates the modification and adjustment of the PAEP and its reliability and validity to measure the level of appropriate admissions and DOS in public pediatric hospitals in Iran.

## 3. Materials and Methods

### 3.1. Cross-Cultural Translation

The tool was translated from English into Persian (9) using the process of cross-cultural translation through the following steps:

1. translation from English to Persian;
2. organizing a working group, including two experienced pediatricians, one methodologist, one English language professional, and one translator to construct the first Persian draft;
3. pilot-testing of the draft on medical records of patients;
4. second meeting of the working group to construct a new consensus version;
5. translating from Persian to English and re-evaluating the instrument by the working group.

### 3.2. Reliability and Validity

The translated PAEP was modified and adjusted in a two-stage process (6). Five physicians (three pediatricians and two general practitioners) made some modifications to the American version of the PAEP to be used in the Iranian context using a nominal group technique. The modified PAEP (Appendix 1) was then used by reviewers (the physicians and researchers [two nurses and the first author]) in a retrospective study in order to examine the inter-rater reliability and external validity of the modified tool.

 This study was performed on 100 case records randomly selected from two public teaching hospitals at Tehran University of Medical Sciences in Iran from 21 November 2012 to March 2013. One of the authors summarized the medical records of the patients in a standardized abstract format (using panel of expert opinion). To safeguard patient’s confidentiality, the standardized abstract format was copied, and patients’ identifications (ID) were deleted. The physicians were identified with an anonymous ID code.

The sample size was calculated considering a disagreement degree of 30% with a 2-tailed confidence interval of ± 10% and 95% confidence. A minimum sample of 84 hospital admissions was calculated with 25% more to compensate for exclusion-associated losses (in total: 105). The patients admitted for elective surgery, burns, intensive care, psychiatric problems, and patients older than 18 years old were excluded.

Before performing the reviews, the reviewers were trained by using the PAEP reviewers’ manual. Then, the reviewers independently and concurrently evaluated medical records. Along with assessing the admission details, the group also assessed 153 DOS in which the patients stayed in the hospitals longer than 48 hours.

Inter-rater reliability was tested by calculating the level of overall agreement and specific agreement between reviewers’ assessments based on the PAEP (three pediatricians, two general practitioners and three researchers). Overall agreement is the proportion of judgments in which two reviewers agree. Specific inappropriate agreement is the proportion of judgments (among those judged to be inappropriate by at least one of the two reviewers) that are rated as being inappropriate by both reviewers. Specific appropriate agreement is also calculated in a similar way (6). In addition, overall agreement between reviewers was evaluated by the kappa (*k*) statistic (10).

In order to test the validity of PAEP, a separate group of clinicians (experienced physicians), including three pediatricians assessed 100 admissions and 153 DOS using individual judgment concerning the appropriateness of the admissions and DOS. The assessments of the three groups of the reviewers based on the PAEP were compared with those of the clinician (11). All the raters (reviewers and clinicians) were asked to judge whether each admission and DOS were appropriate or not. Sensitivity, specificity, positive and negative predictive values of the developed tool was calculated. Experienced clinicians’ judgment was employed as the gold standard in these analyses (12). Finally, Kappa coefficient was also calculated to evaluate the agreement between the reviews using PAEP and the experienced clinicians’ judgment.

Statistical analysis was performed using the Statistical Package for Social Sciences (Windows version 10.0; SPSS Inc. Chicago, United States). Landis and Koch’s guiding principles were employed in interpreting the levels. According to these guidelines, the coefficients between 0.41 and 0.60 are considered as moderate; between 0.61 and 0.80 as substantial, and between 0.81 and 1.00 as perfect (13). The Ethics Committee of Tehran University of Medical Sciences approved this study (on October 3, 2012 with approval No. 90-04-136-16139-97822).

## 4. Results

### 4.1. The Consensus Process

There were no fundamental changes between the American PAEP and its Iranian version (IR-PAEP). Regarding the admission criteria the nominal group made some changes to the criteria of “severity of illnesses.” The criteria 8 and 13, “electrolyte abnormality” and “procedures for which outpatient departments are not responsible” were the major concerns of the group. In criterion 8, the following values were added: “BUN > 45 mg/dL,” “BS ≤ 200-, or BS ≥ 50- mmol/L,” “WBC ≤ 15000, or WBC ≥ 2500,” and in criterion 13, the following sub criteria were added: “unbearable pain,” “abdominal tenderness,” and “foreign body ingestion”. Also, for criterion 9, “hematocrit < 30 %,” and 15, “seizures” were added and “lack of alternative care,” “social acceptance,” and “provision of care in case there is a need for time to take the patient to other centers” are considered.

Regarding DOS criteria, the nominal group agreed to change “nursing/life support services” to “nursing/life support services (where/when no alternative care exists or there is no individual to be trained in order to do any of the procedures at home)”. Finally, group unanimously removed criterion of “IM medication for at least 8 hours that day”. These items are significantly different from the American PAEP.

### 4.2. Reliability and Validity Testing of the Instrument

We selected 105 hospital admissions by a simple random sampling method in which 4.76% of the patients were excluded. Out of 100 patient files, 324 days of stay were obtained. Then the days of admission and discharge were excluded. Those files which lack information referring to the day of clinical-file evaluation (n = 39) or have incomplete notations (n = 32) were also excluded, and 153 days of stay was remained for the study sample.

The reliability in admissions and DOS were almost the same regardless of using override option. In general, the reliability in the samples decreased when the overrides were considered (without overrides = 73.5% and with overrides = 65.5%). Specific inappropriate agreement without overrides was equal to 81.5% and specific inappropriate agreement with overrides was 71%. As there is a possibility for overrides to create bias (6), we avoided using the override option.

The results obtained in this study are shown in the following tables (Tables 1, 2, 3, 4 and 5). Tables 1 and 2 show selected characteristics and distribution of clinical diagnoses for all admissions. Table 3 shows the level of agreement of the reviewers for the IR-PAEP criteria. In general, overall agreements on the assessment of admissions and DOS were very high (96% and 88% respectively) and Cohen’s kappa coefficients (0.75.5 and 0.71, respectively) showed substantial agreement.

There was also a similar level of overall agreements on admissions and DOS among pediatricians (91% and 88%), general practitioners (96% and 91%), and researchers (95% and 94%, respectively). Kappa coefficients showed substantial agreement (0.75 and 0.73) among pediatricians and complete agreement in general practitioners (0.86 and 0.80) and researchers (0.81 and 0.84, respectively).

The findings in Table 3 were compared with the results in Table 4 in which the subjective judgment of the clinicians was regarded as the gold standard. Table 5 shows the results of “sensitivity”, “specificity”, “positive predictive values”, and “negative predictive values” for admissions which were 0.91, 0.85, 0.96, and 0.70 and for DOS were 0.83, 0.92, 0.97, and 0.67, respectively. Cohen’s statistic on admissions and DOS (0.67.3 and 0.61.8, respectively) showed substantial agreement.

The IR-PAEP on admissions had the highest sensitivity (0.93) and specificity (0.88) in researchers’ results and the lowest sensitivity (0.87) and specificity (0.86) in pediatricians’ results. The IR-PAEP on DOS had almost similar sensitivity and specificity in all groups (0.82-0.84 and 0.90-0.91, respectively). Positive and negative predictive values were almost similar in all groups (0.65-0.69.5 and 0.62-0.71, respectively). Kappa coefficients on admissions and DOS showed substantial agreement in pediatricians’ results (0.63.7 and 0.61), general practitioners’ results (0.71 and 0.62), and researchers’ results (0.67.3 and 62.5, respectively).

The overall agreement for reviewers using the IR-PAEP on admissions and DOS was higher (92% and 88%) in comparison with the overall agreement of the clinicians using their subjective judgment (83% and 84%, respectively). Furthermore, the agreement in terms of Kappa coefficient in reliability of the IR-PAEP for reviewers on admissions and DOS was higher (*k* = 0.75.5 and 0.71) in comparison with the reliability of the clinicians using their subjective judgment (*k* = 0.73 and 0.66, respectively).

**Table 1.** Selected Characteristics of the Study Population

	Number
**Age Group, y**	
3 ≥	72
3 <	28
**Gender**	
Male	58
Female	42
**Place of Residence**	
Tehran	60
Non-Tehran	40
**Insurance**	
Yes	82
No	18

**Table 2.** Distribution of Clinical Diagnoses for all Admissions

Variable	Value
**Gastroenteritis**	15
**Seizures and fever**	14
**Urinary tract infection**	13
**Acute upper respiratory infections**	12
**Appendicitis**	8
**Lower respiratory infections**	8
**Cardiac diseases**	7
**Other diagnosis**	23

**Table 3.** Inter-Rater Reliability of the PAEP on Admissions and DOS^a,b^

	Reviewers		Pediatricians		GP		Researchers	
	A	DOS	A	DOS	A	DOS	A	DOS
**Overall agreement, %**	96	88	91	88	96	91	95	94
**SAA, %**	93	89	89	82	95	84	93	91
**SIA, %**	80	83	75	69	83	77	81	86
**Cohen’s k** ** (95% CI for k)**	0.75.5 (0.59-0.94)	0.71 (0.50-0.96)	0.75 (0.66-0.85)	0.73 (0.68-0.79)	0.86	0.80	0.81 (0.72-0.88)	0.84 (0.71-0.96)

^a^ Abbreviations: GP, general practitioners; A, admission; DOS, day of stay; SAA, specific appropriates agreement; SIA, specific inappropriate agreement; CI, confidence interval.

^b^ Reviewers, pediatricians, general practitioners and trained PAEP reviewers (researchers); Researchers, two nurses and the first author; Overrides: for admission = 4.12%, and for DOS = 4.5%, Appropriateness: average appropriate and inappropriate ratings by PAEP reviewers for admission = 70.2% and 23.2%; and for the DOS = 57.7% and 37.8% respectively, P < 0.0001.

**Table 4.** Agreement Among Judgments of Clinicians on Admissions and DOS ^a,b^

	Overall Agreement, %	SAA, %	SIA, %	Cohen’s k 95% CI for k
**Admission**	83	91	70	0.73 (0.67-0.77)
**DOS**	84	89	72	0.66 (0.48-0.90)

^a^ Abbreviations: SAA, specific appropriates agreement; SIA, specific inappropriate agreement; DOS, day of stay; CI, confidence interval.

^b^ P < 0.0001; Uncertainty: clinicians ‘cannot decide’ for admission = 7.1% and for DOS = 6.5%, total appropriate and inappropriate ratings by clinicians for admission = 74.6% and 18.6%; and for DOS = 68% and 25.5% respectively; Disagreement: on admission = 17% and on DOS = 16%.

**Table 5.** Validity of the PAEP When Compared with the Judgments of Clinicians^a,b^

	Raters		Pediatricians		GP		Researchers	
	A	DOS	A	DOS	A	DOS	A	DOS
**Sensitivity**	0.91	0.83	0.87	0.84	0.92	0.84	0.93	0.82
**Specificity**	0.85	0.92	0.86	0.91	0.88	0.94	0.78	0.90
**PPV**	0.96	0.97	0.96	0.96.5	0.96.5	0.97.5	0.94	0.96
**NPV**	0.70	0.67	0.65	0.69	.069	0.69.5	0.68	0.65
**Cohen’s k ** **(95% CI for k)**	0.67.3 (0.51-0.82)	0.61.8 (0.43-0.72)	0.63.7 (0.51-0.82)	0.61 (0.52-0.71)	0.71 (0.69-0.82)	0.62 (0.51-0.75)	0.67.3 (0.62-0.71)	62.5 (0.43-0.72)

^a^ Abbreviations: GP, general practitioners; A, admission; DOS, day of stay; PPV, positive predictive value; NPV, negative predictive value; CI, confidence interval.

^b^ Overall agreement: for admission = 93%; and for DOS = 85.5%, P < 0.0001; Raters, pediatricians, general practitioners, trained PAEP reviewers (researchers) and clinicians; Researchers, two nurses and the first author.

## 5. Discussion

The current study represents the first effort to develop an instrument for measuring the extent of appropriateness of admissions and DOS in pediatric hospitals in the Iranian context. In this research, some criteria were modified and adjusted for admission, including removing the criteria of “intramuscular medication”, and considering “lack of alternative care”, “social acceptance”, and “provision of care in case there is a need for time to take the patient to other centers” which were similar to the UK study (4).

Difference in admission of suspected cases of child seizure is an example of why such cases are routinely admitted to the hospitals in Iran and not in the US (6) and UK (4). In general, the important changes are related to the criteria dealing with “severity of illnesses”.

Also, for DOS criteria, “the need to hospital stay” to be checked and offered paramedical services are considered unacceptable in the Iranian setting except for “interval care” which is similar to the UK study (4). The results of the study showed that the instrument is highly replicable as the agreement between the reviewers on admissions and DOS were 96% and 88% with a *k* statistic of 0.75.5 and 0.71, respectively.

According to the classification of Landis and Koch, *k* statistic value showed a substantial level of reliability. Therefore, non-physicians could be trained to employ the IR-PAEP too. In other words, they will achieve reliable results as physicians. Regarding the admission criteria of the IR-PAEP instrument, level of overall agreement among all reviewers is 96% which is higher than the agreement reported by the developers of the PAEP in the UK (83%) (4). In our study, the level of overall agreement between researchers is 95% which is similar to those reported by the developers of the PAEP in the US (94%) (7) and the UK (96%) (4). Additionally, the level of overall agreement between clinical raters is 83%. This is much higher than those reported by the developers of the PAEP in the UK (59.5%) (4). Furthermore, in this research the overall agreement between physicians and non-physician reviewers is 93%, while, developers in the UK obtained 68% of agreement (8).

Considering the reliability of DOS criteria for the IR-PAEP instrument, the values of *k* statistic among all reviewers is 0.71. However, in the UK, the developers of the PAEP obtained the value of 0.53. The researchers reported a value of 0.84. This value is similar to the value obtained in the UK study (4). According to the results of the study, “sensitivity” and “specificity” values gained were (0.83) and (0.92), respectively. These results are almost comparable to those reported (“sensitivity” = 0.93 and “specificity” = 0.78) in the USA (11). The PAEP was modified and adjusted in the UK by Esmail (4) to be used in pediatric practice. This modified instrument yielded a high level of inter-rater reliability. However, in this study, lack of validation of the instrument using separate specialist panels is obvious.

 In another study in the UK, the PAEP was employed only to the admission criteria and high level of inter-rater reliability was obtained in the modified tool. In the validity exercise by using separate expert panels, the PAEP had limited validity, and it is not recommended for assessment of the UK pediatric practice in general hospitals.

Our results are similar to the North American studies. The similarity and differences of results for validity scores between these countries can be due to differences in payment system to the physicians, whether it is a fee for services or capitation (8).

As high overall agreements can occur with low k scores when the probable prevalence of the factor under investigation is either very high or very low, the decision to employ an instrument should not be made solely on correlation coefficients and rationality. In fact, relevance and suitability of the criteria should be considered together. It is advised that the prevalence of the situation to be measured should not be higher than 50% (14). There is no evidence in the Iranian pediatric hospitals showing the exact rate of inappropriate admission and DOS, but according to the findings of the seven local studies in adults, the percentage of inappropriate hospital admissions and the DOS ranged from 6-22.8% and 6.2-61.2%, respectively (15-21). Therefore, we think that the consequence of the prevalence in this study is not important.

Panel of clinicians is one method to solve the problem as there is no gold standard (12). Panel of clinicians can be considered the ‘the next-best thing’. As a gold standard, it has restrictions since differences between clinicians’ judgments are generally high. In our study, a substantial level of agreement on admissions and DOS was obtained among the members of the clinicians (k = 0. 73 and 0.66 respectively).

When employing the IR-PAEP, important point of concern is the reliability of the protocol when it is used in different sittings (22). In Iran, all public hospitals are centralized and the case mix in various hospitals is not dissimilar. Consequently, the modified version of the protocol is applicable in other hospitals across the country.

The retrospective nature of the study may be mentioned as its major limitation. The results of our study show that the IR-PAEP in its present structure has adequate reliability and validity to measure the extent of appropriateness of admission and DOS. Therefore, it is recommended that the instrument be utilized in pediatric public hospitals in Iran. As the developing countries, particularly Middle East countries have the same status and culture, the result of this study (with minor changes) could be used in these countries.

## Appendix 1.

Pediatric AEP: Admission Criteria (Iranian Version)

A. “Severity of illness” criteria

(1) Sudden onset of unconsciousness (coma or unresponsiveness) or disorientation

(2) Acute or progressive sensory, motor, circulatory or respiratory embarrassment sufficient to incapacitate the patient (inability to move, feed, breathe, urinate, etc.)

(3) Acute loss of sight or hearing

(4) Acute loss of ability to move a main body part

(5) Persistent fever ≥ 37.8°C (100°F) orally or ≥38.3°C, (101°F) rectally

(a) For more than five days

(b) For more than 48 hours when a diagnosis has not been reached

(6) Active bleeding which could lead to circulatory, discomfiture if homeostasis is not protected

(7) Wound dehiscence or evisceration

(8) Severe electrolyte/acid base/CBC abnormality (any of the following values):

(a) or Na ≥ 123 mmol/L, Na ≤ 156 mmol/L

(b) K ≥ 2.5 mmol/L, or K≤ 5.6 mmol/L

(c) HCO_3_ ≥ 14 mmol/L (unless chronically abnormal)

(d) HCO_3_ ≥ 36 mmol/L (unless chronically abnormal)

(e) Arterial pH ≥ 7.45 or Arterial pH ≤ 7.30

(f) Urea > 8 mmol/L or BUN > 45 mg/dL

(j) BS ≥ 50 mmol/L

(h) BS ≥ 200 (associated with symptoms of weight reduction for no apparent reason, polyuria, polydipsia, ketonuria)

(i) WBC ≥ 15000 (ESR ≥ 30)

(k) WBC ≥ 2500 (ANC ≥ 1000)

(9) Hematocrit < 30 % (If is not treatable on an outpatient basis or may be due to the underlying disease)

(10) Pulse more than or less than the following ranges (optimally a lying pulse for under 12 years old child):

1 month to 6 months minus 1 day, 70-170/minute

6 months to 2 years minus 1 day, 80-160/minute

2-6 years, 70-160/minute

7-11 years, 60-160/minute

≥ 12 years, 50-140/minute

(11) BP values outside the following ranges:

6 weeks to 6 months minus 1 day, 70-110 mmHg

(Systolic)

6 months to 2 years minus 1 day, 70-100/40-85 mmHg

2-6 years, 75-125/40-95 mmHg

7-11 years, 80-130/45-90 mmHg

≥ 12 years, 90-150/60-120 mmHg

(12) Need for a lumbar puncture, where this procedure is not done routinely on an outpatient basis

(13) Any of the following procedures not responding to outpatient (including A&E and GP) management:

(a) Cardiac arrhythmia

(b) Bronchial asthma or croup

(c) Dehydration

(d) Continual vomiting or diarrhea which needs advance inpatient assessment

(e) Lack of ability to void or move intestines not due to neurologic disorder

(f) Unbearable pain

(j) Abdominal tenderness which has been examined either in out-patients or by the GP and which requires advance in-patient assessment

(h) Foreign body ingestion (if it is not through the stomach and intestines)

(14) Special pediatric problems:

(a) Child abuse where the severity of injuries necessitates admission or an appropriate protected placement is not available

(b) Non-cooperation with a therapeutic regimen where failure to comply amounts to neglect of the child which puts the child’s immediate health or security at risk

(c) Necessitate special observation or close monitoring of behavior, including calorie intake in cases of failure to thrive

(d) Referred by GP because of lack of ability to manage by the career or any alternatives/social support

(e) Interval care where no alternative exists or the career is not trained to do this at home

(15) Seizures

B. Intensity of service

(1) Surgery or procedure scheduled within 24 hours necessitating;

(a) General or regional anesthesia; or

(b) Use of equipment, facilities or procedure only available in a hospital

(2) Treatment in an intensive care unit

(3) Vital sign monitoring every 2 hours or more often (may include bedside cardiac monitor)

(4) IV medications and/or fluid replacement (does not include tube feeding)

(5) Chemotherapeutic agents that require continuous observation for life-threatening toxic reaction

(6) Intermittent nebulizer use at least every 8 hours

Pediatric AEP: day of care criteria (Iranian version)

A. Medical services

(1) Procedure in the operating room that day, if the procedure is usually done on an inpatient basis in this situation

(2) Procedure scheduled in the operating room within 24 hours, (48 hours for bowel surgery) and which needs preoperative preparation requiring hospital facilities/personnel in 24 hours (48 hours for bowel surgery) prior to the operation

(3) Cardiac catheterization on that day

(4) Angiography, venography or lymphangiography on that day

(5) Invasive diagnostic/therapeutic procedure that day including biopsy of an internal organ (not bone marrow), thoracentesis, paracentesis, cysternal or ventricular tap (not lumbar puncture).

(6) Any test requiring either

(a) Strict dietary control for the duration of the test; or

(b) Collection of a timed sample, lasting 8 hours or more where this cannot be done at home

(7) Documented medical monitoring by physicians at least three separate occasions on that day

B. Nursing/life support services (where/when the career has not been trained to do any of the following procedures at home)

(1) Respiratory care, any respirator use, mist tent, or three or more treatments with inhalation therapy, intermittent positive pressure breathing or chest physical therapy (percussion and drainage)

(2) Parental (intravenous) therapy for at least 8 hours on that day

(3) Continuous monitoring of vital signs or at least every 30 minutes for at least 4 hours or 24 hours after such monitoring

(4) IV medication for at least 8 hours on that day

(5) Strict intake and output measurements and/or calorie counts on that day, under doctor’s orders

(6) Major surgical wound or drainage care (e.g. Chest tubes, tubes, Hamovacs)

(7) Traction for fractures, dislocations, congenital deformities or other orthopedic conditions

(8) Close medical monitoring (vital signs, neurological checks or extremity checks) at least three times daily under doctor’s orders

(9) Interval cares where there is no alternative

C. Patient condition

Within 24 hours of the day reviewed:

(1) Acute inability to void

(2) Transfusion due to acute blood loss

(3) Physician suspicion of suicide attempt so that a psychiatric opinion is requested

(4) Physician suspicion of child abuse or neglect, where suitable alternative placement not available

Within 48 hours of the day reviewed:

(5) Temperature of at least 38.3°C (101°F) rectally or 37.8°C (100°F) orally, if patient admitted for a reason other than fever

(6) Coma; unresponsiveness for at least 1 hour

(7) Acute confusional state

(8) Acute hematological disorder (e.g. Neutropenia, Anemia, thrombocytopenia)

(9) Progressive, acute neurological difficulties
